# MicroRNA expression and its implications for diagnosis and therapy of gallbladder cancer

**DOI:** 10.18632/oncotarget.4227

**Published:** 2015-05-22

**Authors:** Zheng Li, Xin Yu, Jianxiong Shen, Priscilla T.Y. Law, Matthew T.V. Chan, William K.K. Wu

**Affiliations:** ^1^ Department of Orthopaedic Surgery, Peking Union Medical College Hospital, Chinese Academy of Medical Sciences and Peking Union Medical College, Beijing, China; ^2^ Department of Microbiology, The Chinese University of Hong Kong, Hong Kong, China; ^3^ Department of Anaesthesia and Intensive Care and State-Key Laboratory of Digestive Diseases, LKS Institute of Health Sciences, The Chinese University of Hong Kong, Hong Kong, China

**Keywords:** microRNA, gallbladder cancer, proliferation, apoptosis, metastasis

## Abstract

Gallbladder cancer is the most common biliary tract malignancy with poor prognosis. MicroRNAs (miRNAs) are a class of small, endogenous, non-coding RNAs of 19–23 nucleotides in length, which regulate gene expression at post-transcriptional and translational levels. Several studies have demonstrated aberrant expression of miRNAs in gallbladder cancer tissues. Recent evidences also demonstrated that specific miRNAs are functionally involved in gallbladder cancer development through modulating cell proliferation, apoptosis, migration, invasion and metastasis. In this review, we explore the possibilities of using miRNAs as prognostic, diagnostic markers and therapeutic targets in gallbladder cancer.

## INTRODUCTION

Gallbladder cancer is the most common biliary tract malignancy with poor prognosis and the fifth most common gastrointestinal malignancy worldwide [1-3]. The 5-year survival rates of advanced-staged GBC patients ranges from 20% to 40% [4-7]. Gallbladder cancer is usually diagnosed at advanced stage due to absence of specific symptoms [8-10]. Despite recent advances in its diagnostic techniques and therapeutic managements that might give hope on consequent disease remission, prognosis of patients with gallbladder cancer remains poor [11-13]. It is therefore of paramount importance to elucidate its molecular biology, genetic causes, and cellular origin in order to develop novel therapeutic strategies to improve clinical outcome of patients with gallbladder cancer [14-17].

MicroRNAs (miRNAs) are evolutionarily conserved, short, endogenous, single-stranded non-coding RNA molecules [18-24]. Through binding to the 3′-untranslated regions (3′-UTRs) of target mRNAs, miRNAs suppress gene expression by inducing mRNA degradation or inhibiting protein translation depending on the degree of sequence complementarity [25-30]. Aberrant miRNA expressions have been reported in various cancers, such as lung, bladder, gastric, nasopharyngeal, breast, liver and pancreatic cancers [31-41]. Moreover, many studies have indicated that miRNAs can play critical roles in modulating cell proliferation, migration, invasion, apoptosis, radio- and chemosensitivity and cancer stem cell phenotype [42-48]. However, miRNAs expression and their implications for diagnosis and therapy of gallbladder cancer remain elusive. In this review, we focus on miRNAs involved in gallbladder development and discuss the potential use of miRNAs as prognostic biomarkers and treatment strategies for gallbladder cancer.

## FUNCTION AND BIOGENESIS OF MIRNA

Previous studies have delineated the molecular basis of miRNA biogenesis, which consists ofseveral steps [49, 50]. It begins with the transcription of primary miRNA (pri-miRNA), which is several-kilobase-long, from a miRNA gene. The pri-miRNA is then excised into one or more approximately70-nucleotide stem-loop structures to form the miRNA precursors (pre-miRNAs) [51]. Then, the Ran-GTP-dependent nuclear export factorexportin-5 transported pre-miRNA into the cytoplasm, where Dicer processes the pre-miRNA into an approximately 22-nucleotide RNA duplex [52]. Both the mature miRNA strand and its complementary strand are located in the 22-nucleotide RNA duplex [53, 54]. The complementary strand of miRNA is then degraded [55]. By incorporating into the RNA-induced silencing complex (RISC) and binding to the complementary sequences in 3′-UTRs of target mRNAs, miRNA exerts its function through inducing mRNA cleavage or translational inhibition [56, 57].

## MIRNAS IN GALLBLADDER CANCER

The first study on the potential association between genetic variations of miRNA genes and susceptibility to gallbladder cancer was published by Srivastava *et al.* [58]. This case-control study evaluated the potential association of three single nucleotide polymorphisms (SNPs), namelyrs2910164, rs11614913 and rs3746444, with risk for gallbladder cancer in 230 cases and 230 controls in a North Indian population. A non-significant increased risk was observed between carriers of variant genotypes of rs2910164, rs11614913 and rs3746444 (odd ratios=1.3, 1.3 and 1.1, respectively). Their data showed that common miRNA variants may not contribute to gallbladder cancer susceptibility in this population.

The first report on miRNA expression profiling in gallbladder cancer was performed in transgenic BK5.erbB2 mice, in which gallbladder cancer was induced by expressing murine ErbB2 gene under bovine keratin 5 promoter in the basal layer of epithelial tissues by Kitamura *et al*. [59]. MiRNA profiling of 368 miRNAs revealed 9 and 13 significantly upregulated and downregulated miRNAs, respectively, in the gallbladder cancer tissues of these transgenic mice compared with the wide-type mice (>2.2-fold, *p* < 0.05). Furthermore, treatment with histone deacetylase inhibitor PCI-24781 significantly reversed these deregulated miRNAs. For instance, miR-21, miR-142-3p, miR-142-5p, and miR-223, which were upregulated in gallbladder cancer tissue, were decreased upon PCI-24781 treatment. In contrast, miR-122, which was downregulated in gallbladder cancer, was significantly upregulated by PCI-24781, implicating the potential chemotherapeutic value of histone deacetylase inhibition and reversal of miRNA dysregulation in treating biliary tract cancers.

Further studies on miRNAs expression profiling also identified a number of significantly deregulated miRNAs in gallbladder cancer tissues, which are listed in Table 1. For example, Letelier *et al.* [60]. utilized microarray to profile miRNA expression in 6 cancerous and 4 normal gallbladder tissues. Dysregulated miRNAs were further validated by TaqMan RT-PCR in an independent cohort of 8 tumors and 3 non-cancerous gallbladder samples. Through this two-stage approach; the authors confirmed the downregulation of miR-133a, miR-133b, miR-143, miR-145, miR-1, miR-148 and miR-29 c. Pathway enrichment analysis revealed that the most downregulated miRNAs (miR-1, miR-133, miR-143 and miR-145) collectively targeted a number of genes belonging to signaling pathways involved in tumor pathogenesis, such as transforming growth factor (TGF)-β, ErbB3, WNT, vascular endothelial growth factor (VEGF), and those regulating cell motility or adhesion. Functional characterization revealed that miR-1 and miR-145 could significantly inhibit cell viability and induce apoptosis in cultured gallbladder cancer NOZ cells, substantiating their roles as tumor suppressors in gallbladder cancer.

**Table 1:** MiRNA expression profiles in GBC

Num	Method	sample	upregulated	downregulated	Reference
1	PCR-RFLP	primary GBC tissues			58
2	Microarray RT-PCR	mice	miR-21, miR-142-3p, miR-142-5p, miR-223	miR-122	59
3	Microarray RT-PCR	primary GBC		miR-133a miR-133b miR-143-3p miR-145-5p miR-99a-5p miR-125b-5p miR-1 miR-29c-3p miR-195-5p miR-139-5p miR-29c-5p miR-100-5p miR-143-5p miR-148a-3p miR-145-3p miR-376c miR-187-3p miR-365a-3p miR-29b-3p miR-497-5p miR-654-3p miR-411-5p miR-125a-5p miR-26a-5p miR-101-3p miR-495 miR-381-3p miR-154-5p miR-99a-3p miR-328 miR-299-5p miR-30e-3p miR-29b-2-5p miR-379-5p miR-140-5p miR-24-1-5p miR-101-5p	60

Dicer and Drosha are two key enzymes that are involved in the biogenesis of mature miRNAs [61, 62]. The expression of these two genes was detected in 21 non-dysplastic gallbladder epithelia and 108 gallbladder cancer tissues using immunohistochemical staining [55]. It was demonstrated that Dicer and Drosha expression was significantly lower in gallbladder cancer than that in non-dysplastic gallbladder epithelia. The absence or low expression of Dicer or Drosha was also associated with poor differentiation, lymph node metastasis, invasiveness, and failure of radical resection. Univariate Kaplan–Meier analysis showed that the loss of Dicer and Drosha expression was predictive of decreased overall survival independent of other clinic pathological parameters. Taken together, impairment of miRNA biogenesis contributes to metastasis, invasion, and poor prognosis in gallbladder cancer [63].

A number of deregulated miRNAs have been reported to function as tumor suppressors or oncogenes in gallbladder cancer via derepressing important signaling mediators along specific signaling pathways pertinent to cancer development.

## UPREGULATED MIRNAS IN GALLBLADDER CANCER

The biological functions and/or prognostic significance of three upregulated miRNAs, namely miR-20a, miR-155 and miR-182, have been studied in details. MiR-20a was up-regulated in gallbladder cancer tissues as demonstrated by both real-time RT-PCR and *in situ* hybridization. Overexpression of miR-20a promoted invasion and proliferation of gallbladder cancer cells, accompanied by dysregulation of several epithelial–mesenchymal transition-related genes *in vitro* and *in vivo*. Smad7 (mothers against decapentaplegic homolog 7), a potential inhibitor of TGF-β1 signaling pathway, was identified as the direct target of miR-20a. Clinically, patients with higher miR-20a levels exhibited worse overall survival [64]. Kono *et al*. reported that miR-155 was significantly overexpressed in gallbladder cancer when compared with gallbladders with pancreaticobiliary maljunction and normal gallbladders [65]. The high expression level of miR-155 in gallbladder cancer was significantly associated with the presence of lymph node metastasis and poor prognosis. *In vitro* assays showed that aberrant expression of miR-155 significantly enhanced gallbladder cancer cell proliferation and invasion. Qiu *et al*. found that miR-182 levels was significantly upregulated in GBC tissues compared with normal controls, and miR-182 expression was remarkably increased in primary tumors that subsequently metastasized, when compared to those non-metastatic tumors [66]. Interestingly, TGF-β induced miR-182 expression in gallbladder cancer cells whereas overexpression of miR-182 promoted gallbladder cell migration and invasion. Importantly, miR-182 inhibition suppressed TGF-β-induced cancer cell migration and invasion. Blockade of miR-182 also effectively inhibited pulmonary metastases *in vivo*. Cell adhesion molecule1 (CADM1) was further identified as a new target of miR-182. In this regard, miR-182 inhibited CADM1 expression *in vitro* and *in vivo*.

## DOWNREGULATED MIRNAS IN GALLBLADDER CANCER

In stark contrast to the scarcity of reported upregulated miRNAs in gallbladder cancer, miRNA downregulation is more pervasive, suggestive of the tumor-suppressing function of miRNAs as a whole. MiR-335 levels was one of the most significantly under-expressed miRNAs in gallbladder cancer, in which 58% cases exhibited downregulation when compared with their adjacent nondysplastic counterparts as measured by RT-PCR [67]. Clinic pathological correlation further showed that miR-335 expression was significantly lower in gallbladder tissues with high histological grade, advanced pathologic T (tumor) and clinical stages, and lymph node metastasis. Univariate and multivariate analyses further revealed that miR-335 levels could serve as an independent prognostic marker for overall survival. MiR-34a is another hallmark downregulated miRNA in gallbladder cancer. Jin *et al.* measured miR-34a expression and telomere length in 77 gallbladder cancer tissues and 36 peritumoral tissues [68]. Significant downregulation of miR-34a and longer telomere length were observed in gallbladder cancer tissues, in which such alterations were correlated with poor prognosis. Mechanistically, restored expression of miR-34a inhibited the colony-forming ability of CD44^+^CD133^+^ gallbladder cancer stem-like cells *in vitro* and the growth of tumor xenograft *in vivo*. Adenovirus-mediated expression of miR-34a could also downregulate PNUTS, a protein that prevents telomere shortening, and reduce telomere length in gallbladder cancer xenograft. Aside from miR-335 and miR-34a, miR-130a was markedly downregulated in gallbladder cancer tissues in which miRNA-130a levels were negatively correlated with HOTAIR, a trans-regulatory long noncoding RNA (lncRNA) [69]. The authors reported that knockdown of HOTAIR inhibited the invasion of gallbladder cancer cells while miRNA-130a inhibitor reversed the decrease in invasiveness. Knockdown of HOTAIR also suppressed cancer cell proliferation as manifested as the reduction of S-phase fraction while miRNA-130a inhibitor rescued the proliferation. These results implied that the oncogenic effect of HOTAIR was partly mediated through negative regulation of miRNA-130a. Zhou *et al.* reported that miR-26a [70] and miR-135a-5p [71] levels were significantly reduced in primary gallbladder cancer tissues and their downregulation were associated with poor histological grades. Reintroduction of miR-26a significantly inhibited cell proliferation through induction of G_1_/S cell cycle arrest. Furthermore, high mobility group AT-hook 2 (HMGA2) was found to be the direct target of miR-26a in gallbladder cancer in which HMGA2 mRNA levels and miR-26a levels were negatively correlated. Similar to miR-26a, re-expression of miRNA-135a-5p inhibited gallbladder cancer cell proliferation *in vitro* and *in vivo*, with induction of G_1_/S cell cycle arrest and upregulation of caspase 3/7 activities. Luciferase reporter assay further demonstrated that miR-135a-5p repressed cell proliferation through direct targeting of very-low-density lipoprotein receptor (VLDLR) and repressed p38 MAPK pathway. Ma *et al.* [72] reported that overexpression of CCAT1, a lncRNA, in gallbladder cancer contributed to upregulation of Bmi1, which is the target of miRNA-218-5p. Subsequent analysis confirmed that CCAT1 decreased the availability of miRNA-218-5p by functioning as a ‘miRNA sponge’. These data revealed that CCAT1 enhanced the proliferation and invasiveness of gallbladder cancer cells, at least in part, through disrupting miRNA-218-5p-mediated downregulation of Bmi1. Moreover, CCAT1 transcript levels were correlated with that of Bmi1 in gallbladder cancer tissues. Aquaporins (ACQs) are important in controlling bile formation and could exert oncogenic action if overexpressed in gallbladder cancer. AQP-5 silencing by siRNA restored the expression of miR-29b, -200a, and -21 in gallbladder cancer cells, suggesting the downregulation of these miRNAs might mediate the oncogenic action of AQPs [73] (Table 2).

**Table 2:** Functional characterization of the deregulated miRNAs in GBC

Name	Up or down regulation	Target gene	role	Reference
miR-20a	Up	Smad7	oncogene	64
miR-155	Up		oncogene	65
miR-335	Down		Tumor suppressor	66
miR-29b	Up		oncogene	67
miR-200a	Up		oncogene	67
miR-21	Up	PTEN	oncogene	67
miR-34a	Down	PNUTS	Tumor suppressor	68
miR-130a	Down	HOTAIR	Tumor suppressor	69
miR-182	Up	CADM1	oncogene	70
miR-26a	Down	HMGA2	Tumor suppressor	71
miR-135a-5p	Down	VLDLR	Tumor suppressor	72
miRNA-218-5p	Down	Bmi1	Tumor suppressor	73

## CONCLUDING REMARKS AND FUTURE PERSPECTIVES

Increasing evidence has confirmed the importance of miRNA dysregulation in the progression and pathogenesis of human malignancies including gallbladder cancer. The functional roles of specific miRNAs as oncogenes or tumor suppressors render them attractive targets for therapeutic intervention. Nevertheless, with more research efforts put forth to the development of miRNA-based therapeutics and delivery system, it is hopeful that miRNAs will achieve clinical utility at last.

## References

[1] Bal MM, Ramadwar M, Deodhar K, Shrikhande S (2015). Pathology of Gallbladder Carcinoma: Current Understanding and New Perspectives. Pathology oncology research : POR.

[2] Ha TY, Yoon YI, Hwang S, Park YJ, Kang SH, Jung BH, Kim WJ, Sin MH, Ahn CS, Moon DB, Song GW, Jung DH, Lee YJ, Park KM, Kim KH, Lee SG (2015). Effect of Reoperation on Long-Term Outcome of pT1b/T2 Gallbladder Carcinoma After Initial Laparoscopic Cholecystectomy. Journal of gastrointestinal surgery : official journal of the Society for Surgery of the Alimentary Tract.

[3] Doval DC, Azam S, Sinha R, Batra U, Mehta A (2014). Expression of epidermal growth factor receptor, p53, Bcl2, vascular endothelial growth factor, cyclooxygenase-2, cyclin D1, human epidermal receptor-2 and Ki-67: Association with clinicopathological profiles and outcomes in gallbladder carcinoma. Journal of carcinogenesis.

[4] Yu S, Yang Q, Yang JH, Du Z, Zhang G (2015). Identification of suitable reference genes for investigating gene expression in human gallbladder carcinoma using reverse transcription quantitative polymerase chain reaction. Molecular medicine reports.

[5] Chapman BC, Jones T, McManus MC, Shah R, Gajdos C (2014). Metastatic papillary gallbladder carcinoma with a unique presentation and clinical course. JOP : Journal of the pancreas.

[6] Moy AP, Shahid M, Ferrone CR, Borger DR, Zhu AX, Ting D, Deshpande V (2015). Microsatellite instability in gallbladder carcinoma. Virchows Archiv : an international journal of pathology.

[7] Zhang LQ, Xu XS, Wan Y, Song SD, Wang RT, Chen W, Wang ZX, Chang HL, Wei JC, Dong YF, Liu C (2015). Prognostic implications of estrogen receptor 1 and vascular endothelial growth factor A expression in primary gallbladder carcinoma. World journal of gastroenterology : WJG.

[8] Huang L, Chen W, Liang P, Hu W, Zhang K, Shen S, Chen J, Zhang Z, Chen B, Han Y, Meng F, DeMorrow S, Yin X, Lai J, Liang L (2015). Serum CYFRA 21-1 in Biliary Tract Cancers: A Reliable Biomarker for Gallbladder Carcinoma and Intrahepatic Cholangiocarcinoma. Digestive diseases and sciences.

[9] Cui HX, Ma XD, Han XL, Zhang XH (2014). Surgical strategies for unexpected gallbladder carcinoma. European review for medical and pharmacological sciences.

[10] Kijima H, Wu Y, Yosizawa T, Suzuki T, Tsugeno Y, Haga T, Seino H, Morohashi S, Hakamada K (2014). Pathological characteristics of early to advanced gallbladder carcinoma and extrahepatic cholangiocarcinoma. Journal of hepato-biliary-pancreatic sciences.

[11] Weng H, Tan ZJ, Hu YP, Shu YJ, Bao RF, Jiang L, Wu XS, Li ML, Ding Q, Wang XA, Xiang SS, Li HF, Cao Y, Tao F, Liu YB (2014). Ursolic acid induces cell cycle arrest and apoptosis of gallbladder carcinoma cells. Cancer cell international.

[12] Igami T, Ebata T, Yokoyama Y, Sugawara G, Mizuno T, Yamaguchi J, Shimoyama Y, Nagino M (2015). Combined Extrahepatic Bile Duct Resection for Locally Advanced Gallbladder Carcinoma: Does It Work?. World journal of surgery.

[13] Yun SP, Shin N, Seo HI (2015). Clinical outcomes of small cell neuroendocrine carcinoma and adenocarcinoma of the gallbladder. World journal of gastroenterology : WJG.

[14] Willekens I, Goethals LR, Brussaard C, Verdries D, de Mey J (2014). Correlative imaging in gallbladder carcinoma. JBR-BTR.

[15] Abdelilah B, Mohamed O, Yamoul R, Elkhiyat I, Al Bouzidi A, Alkandry S, Abdelkader E (2014). Acute cholecystitis as a rare presentation of metastatic breast carcinoma of the gallbladder: A case report and review of the literature. The Pan African medical journal.

[16] Weng M, Zhang M, Qin Y, Gong W, Tang Z, Quan Z, Wu K (2014). Targeting gallbladder carcinoma: bone marrow-derived stem cells as therapeutic delivery vehicles of myxoma virus. Chinese medical journal.

[17] Tao J, Xu XS, Song YZ, Qu K, Wu QF, Wang RT, Meng FD, Wei JC, Dong SB, Zhang YL, Tai MH, Dong YF, Wang L, Liu C (2014). Down-regulation of FoxM1 inhibits viability and invasion of gallbladder carcinoma cells, partially dependent on inducement of cellular senescence. World journal of gastroenterology : WJG.

[18] Li Z, Yu X, Shen J, Jiang Y (2015). MicroRNA dysregulation in uveal melanoma: a new player enters the game. Oncotarget.

[19] Li Z, Yu X, Shen J, Wu WK, Chan MT (2015). MicroRNA expression and its clinical implications in Ewing's sarcoma. Cell proliferation.

[20] Yu X, Li Z (2014). MicroRNAs regulate vascular smooth muscle cell functions in atherosclerosis (review). International journal of molecular medicine.

[21] Yu X, Li Z, Shen J, Wu WK, Liang J, Weng X, Qiu G (2013). MicroRNA-10b Promotes Nucleus Pulposus Cell Proliferation through RhoC-Akt Pathway by Targeting HOXD10 in Intervetebral Disc Degeneration. PloS one.

[22] Wang Z, Wang N, Liu P, Chen Q, Situ H, Xie T, Zhang J, Peng C, Lin Y, Chen J (2014). MicroRNA-25 regulates chemoresistance-associated autophagy in breast cancer cells, a process modulated by the natural autophagy inducer isoliquiritigenin. Oncotarget.

[23] Hayes J, Thygesen H, Droop A, Hughes TA, Westhead D, Lawler SE, Wurdak H, Short SC (2015). Prognostic microRNAs in high-grade glioma reveal a link to oligodendrocyte precursor differentiation. Oncoscience.

[24] Sidiropoulos KG, White NM, Bui A, Ding Q, Boulos P, Pampalakis G, Khella H, Samuel JN, Sotiropoulou G, Yousef GM (2014). Kallikrein-related peptidase 5 induces miRNA-mediated anti-oncogenic pathways in breast cancer. Oncoscience.

[25] Bier A, Giladi N, Kronfeld N, Lee HK, Cazacu S, Finniss S, Xiang C, Poisson L, de Carvalho AC, Slavin S, Jacoby E, Yalon M, Toren A, Mikkelsen T, Brodie C (2013). MicroRNA-137 is downregulated in glioblastoma and inhibits the stemness of glioma stem cells by targeting RTVP-1. Oncotarget.

[26] Heo MJ, Kim YM, Koo JH, Yang YM, An J, Lee SK, Lee SJ, Kim KM, Park JW, Kim SG (2014). microRNA-148a dysregulation discriminates poor prognosis of hepatocellular carcinoma in association with USP4 overexpression. Oncotarget.

[27] Zhang C, Liu J, Wang X, Wu R, Lin M, Laddha SV, Yang Q, Chan CS, Feng Z (2014). MicroRNA-339-5p inhibits colorectal tumorigenesis through regulation of the MDM2/p53 signaling. Oncotarget.

[28] Huang HJ, Liu J, Hua H, Li SE, Zhao J, Yue S, Yu TT, Jin YC, Cheng SY (2014). MiR-214 and N-ras regulatory loop suppresses rhabdomyosarcoma cell growth and xenograft tumorigenesis. Oncotarget.

[29] Luzhna L, Kovalchuk O (2014). Low dose irradiation profoundly affects transcriptome and microRNAme in rat mammary gland tissues. Oncoscience.

[30] Ninio-Many L, Grossman H, Levi M, Zilber S, Tsarfaty I, Shomron N, Tuvar A, Chuderland D, Stemmer SM, Ben-Aharon I, Shalgi R (2014). MicroRNA miR-125a-3p modulates molecular pathway of motility and migration in prostate cancer cells. Oncoscience.

[31] Li Z, Yu X, Wang Y, Shen J, Wu WK, Liang J, Feng F (2014). By downregulating TIAM1 expression, microRNA-329 suppresses gastric cancer invasion and growth. Oncotarget.

[32] Li Z, Lei H, Luo M, Wang Y, Dong L, Ma Y, Liu C, Song W, Wang F, Zhang J, Shen J, Yu J (2015). DNA methylation downregulated mir-10b acts as a tumor suppressor in gastric cancer. Gastric cancer : official journal of the International Gastric Cancer Association and the Japanese Gastric Cancer Association.

[33] Ohdaira H, Sekiguchi M, Miyata K, Yoshida K (2012). MicroRNA-494 suppresses cell proliferation and induces senescence in A549 lung cancer cells. Cell proliferation.

[34] Fei B, Wu H (2013). MiR-378 inhibits progression of human gastric cancer MGC-803 cells by targeting MAPK1 *in vitro*. Oncology research.

[35] Zhang WH, Gui JH, Wang CZ, Chang Q, Xu SP, Cai CH, Li YN, Tian YP, Yan L, Wu B (2012). The identification of miR-375 as a potential biomarker in distal gastric adenocarcinoma. Oncology research.

[36] Zhou J, Song S, Cen J, Zhu D, Li D, Zhang Z (2012). MicroRNA-375 is downregulated in pancreatic cancer and inhibits cell proliferation *in vitro*. Oncology research.

[37] Liang J, Zhang Y, Jiang G, Liu Z, Xiang W, Chen X, Chen Z, Zhao J (2013). MiR-138 induces renal carcinoma cell senescence by targeting EZH2 and is downregulated in human clear cell renal cell carcinoma. Oncology research.

[38] Furuta M, Kozaki K, Tanimoto K, Tanaka S, Arii S, Shimamura T, Niida A, Miyano S, Inazawa J (2013). The tumor-suppressive miR-497-195 cluster targets multiple cell-cycle regulators in hepatocellular carcinoma. PloS one.

[39] Zeng X, Xiang J, Wu M, Xiong W, Tang H, Deng M, Li X, Liao Q, Su B, Luo Z, Zhou Y, Zhou M, Zeng Z, Shen S, Shuai C, Li G (2012). Circulating miR-17, miR-20a, miR-29c, and miR-223 combined as non-invasive biomarkers in nasopharyngeal carcinoma. PloS one.

[40] Donzelli S, Strano S, Blandino G (2014). microRNAs: short non-coding bullets of gain of function mutant p53 proteins. Oncoscience.

[41] Li H, Gupta S, Du WW, Yang BB (2014). MicroRNA-17 inhibits tumor growth by stimulating T-cell mediated host immune response. Oncoscience.

[42] Schirmer U, Doberstein K, Rupp AK, Bretz NP, Wuttig D, Kiefel H, Breunig C, Fiegl H, Muller-Holzner E, Zeillinger R, Schuster E, Zeimet AG, Sultmann H, Altevogt P (2014). Role of miR-34a as a suppressor of L1CAM in endometrial carcinoma. Oncotarget.

[43] Li M, Yu M, Liu C, Zhu H, He X, Peng S, Hua J (2013). miR-34c works downstream of p53 leading to dairy goat male germline stem-cell (mGSCs) apoptosis. Cell proliferation.

[44] Li J, You T, Jing J (2014). MiR-125b inhibits cell biological progression of Ewing's sarcoma by suppressing the PI3K/Akt signalling pathway. Cell proliferation.

[45] Huang J, Zhang SY, Gao YM, Liu YF, Liu YB, Zhao ZG, Yang K (2014). MicroRNAs as oncogenes or tumour suppressors in oesophageal cancer: potential biomarkers and therapeutic targets. Cell proliferation.

[46] Chiang Y, Zhou X, Wang Z, Song Y, Liu Z, Zhao F, Zhu J, Xu H (2012). Expression levels of microRNA-192 and -215 in gastric carcinoma. Pathology oncology research : POR.

[47] Gurung B, Katona BW, Hua X (2014). Menin-mediated regulation of miRNA biogenesis uncovers the IRS2 pathway as a target for regulating pancreatic beta cells. Oncoscience.

[48] Sanchez-Diaz PC, Hsiao TH, Zou Y, Sugalski AJ, Heim-Hall J, Chen Y, Langevin AM, Hung JY (2014). In silico functional analyses and discovery of survival-associated microRNA signatures in pediatric osteosarcoma. Oncoscience.

[49] Azmi AS, Beck FW, Bao B, Mohammad RM, Sarkar FH (2011). Aberrant epigenetic grooming of miRNAs in pancreatic cancer: a systems biology perspective. Epigenomics.

[50] He JF, Luo YM, Wan XH, Jiang D (2011). Biogenesis of MiRNA-195 and its role in biogenesis, the cell cycle, and apoptosis. Journal of biochemical and molecular toxicology.

[51] Tivnan A, McDonald KL (2013). Current progress for the use of miRNAs in glioblastoma treatment. Molecular neurobiology.

[52] Fabbri M, Calin GA (2010). Epigenetics and miRNAs in human cancer. Advances in genetics.

[53] Wang M, Chu H, Li P, Yuan L, Fu G, Ma L, Shi D, Zhong D, Tong N, Qin C, Yin C, Zhang Z (2012). Genetic variants in miRNAs predict bladder cancer risk and recurrence. Cancer research.

[54] Song B, Ju J (2010). Impact of miRNAs in gastrointestinal cancer diagnosis and prognosis. Expert reviews in molecular medicine.

[55] Bianchi N, Zuccato C, Finotti A, Lampronti I, Borgatti M, Gambari R (2012). Involvement of miRNA in erythroid differentiation. Epigenomics.

[56] Dassow H, Aigner A (2013). MicroRNAs (miRNAs) in colorectal cancer: from aberrant expression towards therapy. Current pharmaceutical design.

[57] Pallasch CP, Patz M, Park YJ, Hagist S, Eggle D, Claus R, Debey-Pascher S, Schulz A, Frenzel LP, Claasen J, Kutsch N, Krause G, Mayr C, Rosenwald A, Plass C, Schultze JL (2009). miRNA deregulation by epigenetic silencing disrupts suppression of the oncogene PLAG1 in chronic lymphocytic leukemia. Blood.

[58] Srivastava K, Srivastava A, Mittal B (2010). Common genetic variants in pre-microRNAs and risk of gallbladder cancer in North Indian population. Journal of human genetics.

[59] Kitamura T, Connolly K, Ruffino L, Ajiki T, Lueckgen A, DiGiovanni J, Kiguchi K (2012). The therapeutic effect of histone deacetylase inhibitor PCI-24781 on gallbladder carcinoma in BK5.erbB2 mice. Journal of hepatology.

[60] Letelier P, Garcia P, Leal P, Alvarez H, Ili C, Lopez J, Castillo J, Brebi P, Roa JC (2014). miR-1 and miR-145 act as tumor suppressor microRNAs in gallbladder cancer. International journal of clinical and experimental pathology.

[61] Van Wynsberghe PM, Chan SP, Slack FJ, Pasquinelli AE (2011). Analysis of microRNA expression and function. Methods Cell Biol.

[62] Vaksman O, Hetland TE, Trope CG, Reich R, Davidson B (2012). Argonaute, Dicer, and Drosha are up-regulated along tumor progression in serous ovarian carcinoma. Human pathology.

[63] Shu GS, Yang ZL, Liu DC (2012). Immunohistochemical study of Dicer and Drosha expression in the benign and malignant lesions of gallbladder and their clinicopathological significances. Pathology, research and practice.

[64] Chang Y, Liu C, Yang J, Liu G, Feng F, Tang J, Hu L, Li L, Jiang F, Chen C, Wang R, Yang Y, Jiang X, Wu M, Chen L, Wang H (2013). MiR-20a triggers metastasis of gallbladder carcinoma. Journal of hepatology.

[65] Kono H, Nakamura M, Ohtsuka T, Nagayoshi Y, Mori Y, Takahata S, Aishima S, Tanaka M (2013). High expression of microRNA-155 is associated with the aggressive malignant behavior of gallbladder carcinoma. Oncology reports.

[66] Qiu Y, Luo X, Kan T, Zhang Y, Yu W, Wei Y, Shen N, Yi B, Jiang X (2014). TGF-beta upregulates miR-182 expression to promote gallbladder cancer metastasis by targeting CADM1. Molecular bioSystems.

[67] Peng HH, Zhang YD, Gong LS, Liu WD, Zhang Y (2013). Increased expression of microRNA-335 predicts a favorable prognosis in primary gallbladder carcinoma. OncoTargets and therapy.

[68] Jin K, Xiang Y, Tang J, Wu G, Li J, Xiao H, Li C, Chen Y, Zhao J (2014). miR-34 is associated with poor prognosis of patients with gallbladder cancer through regulating telomere length in tumor stem cells. Tumour biology : the journal of the International Society for Oncodevelopmental Biology and Medicine.

[69] Ma MZ, Li CX, Zhang Y, Weng MZ, Zhang MD, Qin YY, Gong W, Quan ZW (2014). Long non-coding RNA HOTAIR, a c-Myc activated driver of malignancy, negatively regulates miRNA-130a in gallbladder cancer. Molecular cancer.

[70] Zhou H, Guo W, Zhao Y, Wang Y, Zha R, Ding J, Liang L, Hu J, Shen H, Chen Z, Yin B, Ma B (2014). MicroRNA-26a acts as a tumor suppressor inhibiting gallbladder cancer cell proliferation by directly targeting HMGA2. International journal of oncology.

[71] Zhou H, Guo W, Zhao Y, Wang Y, Zha R, Ding J, Liang L, Yang G, Chen Z, Ma B, Yin B (2014). MicroRNA-135a acts as a putative tumor suppressor by directly targeting very low density lipoprotein receptor in human gallbladder cancer. Cancer science.

[72] Ma MZ, Chu BF, Zhang Y, Weng MZ, Qin YY, Gong W, Quan ZW (2015). Long non-coding RNA CCAT1 promotes gallbladder cancer development via negative modulation of miRNA-218-5p. Cell death & disease.

[73] Sekine S, Shimada Y, Nagata T, Sawada S, Yoshioka I, Matsui K, Moriyama M, Omura T, Osawa S, Shibuya K, Hashimoto I, Watanabe T, Hojo S, Hori R, Okumura T, Yoshida T (2013). Role of aquaporin-5 in gallbladder carcinoma. European surgical research Europaische chirurgische Forschung Recherches chirurgicales europeennes.

